# Methods of Orthodontic Microimplant Surface Modifications Providing Antibacterial Properties: A Systematic Review

**DOI:** 10.3390/ma18153575

**Published:** 2025-07-30

**Authors:** Alicja Wądołowska, Joanna Lis, Beata Kawala, Anna Ewa Kuc, Gabriela Zdrodowska, Agnieszka Rożdżestwieńska-Sowa, Michał Sarul

**Affiliations:** 1 Department of Dentofacial Orthopedics and Orthodontics, Wroclaw Medical University, Krakowska 26, 50-425 Wroclaw, Poland; 2 Department of Integrated Dentistry, Wroclaw Medical University, 50-425 Wroclaw, Poland

**Keywords:** orthodontics, microimplant, orthodontic screw, antimicrobial, coating

## Abstract

The use of orthodontic microimplants in daily practice is now an indispensable part of orthodontic treatment. Unfortunately, the use of skeletal anchorage is associated with a relatively high risk of loss of microimplant stability because of inflammation developing in the surrounding soft tissues. The aim of this systematic review is to identify possible methods of orthodontic microimplant surface modifications providing antibacterial properties. The PubMed, Web of Science, Embase, and Cochrane Reviews databases were searched, and a literature review was conducted. The search was performed between 1 December 2024 and 31 December 2024. The authors used the PICO format to facilitate the search of abstracts and ensure that the relevant components of the question are well defined. The systematic review was written based on the principles detailed in PRISMA. The quality of the papers was assessed based on a tool developed by the authors. Three papers were rated Low Risk of Bias (RoB), four were rated Moderate RoB, and three were rated High RoB. All of the studies presented a method to increase the antibacterial properties of microimplants. More research with a unified methodology is necessary to confirm the effectiveness of the analyzed methods. ZnO, antibiotics, chlorhexidine, silver compounds, selenium, HA, and PEG polymerization plasma represent an interesting perspective for improving the antibacterial properties of orthodontic microimplants.

## 1. Introduction

The use of skeletal anchorage in the form of orthodontic microimplants has revolutionized orthodontic treatment. Temporary Intraoral Skeletal Anchorage Devices (TISADs) have enabled previously difficult dental rearrangements, such as alignment of the occlusal plane and intrusion of lateral sections of the dentition [1]. Unfortunately, their use in daily practice carries the risk of complications, which both doctor and patient need to know about before starting a treatment. These include peri-implantitis, defined as chronic progressive marginal inflammation occurring in tissues surrounding a microimplant [2]. The main symptoms of such a condition are hyperemia, hemorrhage, or proliferation of surrounding soft tissue, and even loosening and falling out of the implant because of the destruction of the implantation site [2,3]. Development of inflammation may also be influenced by the fact that the area of implantation is the most difficult for the patient to clean during brushing. According to the study published by de Freitas et al. [4], it is important to monitor microbial colonization in the implantation site, as conditions favorable for bacterial growth, including poor mini-implant hygiene, may lead to peri-implant infection.

While peri-miniscrew inflammation is a process influenced by many factors, bacterial infection appears to be one of the main causes responsible for this condition [5]. Attempts are underway to develop ways to reduce the risk of this complication, and to do so, it seems essential to understand the factors that affect it. Zhao et al. [5] tried to identify the specific bacteria responsible for peri-miniscrew inflammation. It was shown that around microimplants that failed, there was a stronger correlation with periodontal disease-associated bacteria, such as *Fusobacterium nucleatum*, *Filifactor alocis*, *Porphyromonas gingivalis*, and *Prevotella nigrescens*. The study by Huang et al. [6] demonstrated that in plaque found in the subgingival area around TISADs, the proportion of spirochetes was greater than that in the supragingival plaque, which may also contribute to the destructive effect on the surrounding soft tissues. The study by Garcez et al. [7] proved that a higher number of *Porphyromonas gingivalis* contamination was found around inflamed microimplants. According to the study published by Apel et al. [8], the absence of species that are markers of periodontal health, like *Actinomyces viscosus* and *Cylindrotheca gracilis* in failed microimplants, could be interpreted as a first symptom of a changing microflora, finally leading to peri-implantitis.

It seems no less important to understand the mechanism of inflammation around TISADs. According to the findings of He et al. [2], TLR-2, TLR-4, LOX-1, and BMP participate in the regulation of ILs (IL-1β, IL-6, IL-8, and IL-17), TNF-α, RANKL, MMP-2, and MMP-9 expression via JNK, Erk1/2, Wnt5a, NF-kBp65, OPN, and TAB/TAK signaling pathway, and among them IL-1 beta and IL-6 are critical inflammation mediators in the signaling pathways inducing inflammatory reactions surrounding implants. The discovery may, in the future, allow for better-targeted therapy focused on eliminating a specific factor.

Many attempts have been made to date to develop methods to reduce inflammation around the mini-implants and thus the frequency of complications in the form of reduced stability and loss of TISADs. These include gels and rinses containing antiseptics in their composition [9,10], possibly in combination with the use of irrigators, or the use of photobiomodulation therapy, whose effectiveness was proven in a systematic review published by Zhang et al. [11]. Another method to reduce inflammation around TISADs is to modify the surface of the microimplants by coating them with a material with antibacterial properties. This systematic review attempts to identify methods described to date for modifying the surface of microimplants to enhance their antibacterial properties.

## 2. Materials and Methods

The systematic review was written based on the principles detailed in Preferred Reporting Items for Systematic Reviews and Meta-Analyses (PRISMA Checklist in Supplementary Materials).

### 2.1. Questions

To pose the right question, facilitate the search for abstracts, and make sure that the relevant components of the question are well defined, the authors used the PICO format:

P (patient/population): in vivo studies involving humans or animals, in vitro studies, both concerning implants used for orthodontic treatment;

I (intervention): orthodontic implant modification causing antibacterial effect;

C (comparison): control group, without modification;

O (outcome): influence on the quantity of bacteria around orthodontic implants.

### 2.2. Study Identification and Search Method

An attempt was made to find a systematic review published to date on the studied topic. Such a study has not been found. The search began by identifying keyword combinations: orthodontic, implants, screw, inflammation, coating, antibacterial, antimicrobial, and bacteria.

The search was performed between 1 and 31 December 2024. The PubMed, Web of Science, Embase, and Cochrane Reviews databases were searched, and a literature review was conducted. Articles published in journals between 2003 (the appearance of the entry orthodontic microimplant in Angle Orthodontist for the first time) and 2023 were reviewed. This search yielded 3228 abstracts. Internal and external duplicates were removed, resulting in 2468 papers.

### 2.3. Eligibility Criteria

All the abstracts were analyzed for suitability for further review by two independent authors. Articles that were literature reviews and case reports were excluded. Studies using dental implants were also excluded because their use and mechanical properties differ from those desired for orthodontic microimplants. Studies in which the authors used plates or other materials instead of orthodontic microimplants were not eligible for the review. Studies without a control group were also excluded.

### 2.4. Study Selection

**Figure 1:**
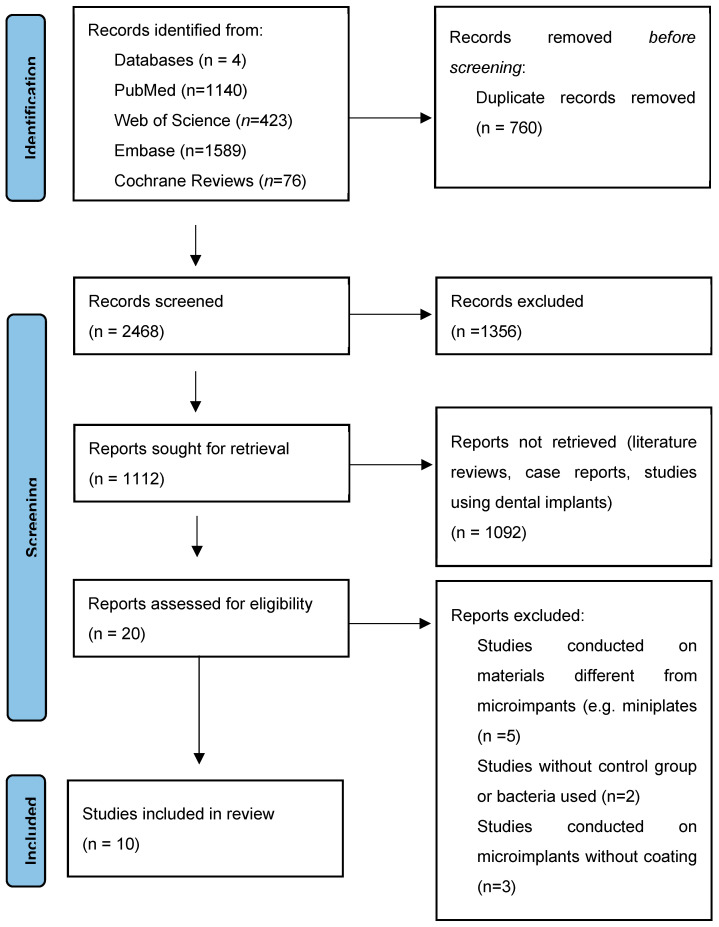
Flow chart.

After final verification of the results, 10 studies were qualified for further analysis. They all turned out to be in vitro tests. All selected studies were published after 2017 (Figure 1).

### 2.5. Risk of Bias Assessment

An attempt was then made to find a suitable tool that could be used to assess the Risk of Bias (RoB). Unfortunately, despite the search, it was not possible to find a tool that fully matched the subject matter of the research. Such difficulties were also identified by Tran et al. [12]. Given the results of their work, after reviewing the methodology of the studies qualified for review, the authors decided to develop a proprietary analysis, which included questions collected from various studies and those developed by the authors.

### 2.6. Data Extraction and Data Synthesis

Each study was analyzed by all authors of the paper, independently. A table was created to include information obtained from the studies (Table 1). A meta-analysis of the obtained results was then attempted.

## 3. Results

The results obtained are summarized in Table 1. As the lack of a control group was an exclusion criterion, control groups were not included in the table.

### Risk of Bias Assessment

In the next step, the authors conducted a Risk of Bias assessment of the results obtained to assess the quality of the studies. The analysis was performed according to the criterion developed from the subject analysis, as well as using questions from other analyses. This yielded 16 questions divided into three sections: Introduction, Methods, and Results (Table 2).

The authors divided the studies into three groups: Low, Moderate, and High Risk of Bias. The summary and description of the most important factors influencing the results are presented in Table 3.

Due to the excessive diversity of studies, especially in terms of methodology, it was not possible to conduct a meta-analysis.

## 4. Discussion

One of the most widely studied methods to modify the surface of microimplants has been the use of ZnO to impart antimicrobial properties. It was included in four studies by Bahrami [21], Abo-Elmahasen [19], Noorollahian [17], and Othman [22]. Efficacy against *Porphyromonas gingivalis* [17,21], *Prevotella intermedia* [21], *Aggregatibacter actinomycetemcomitans* [21], *Enterobacter aeruginosa* [19], *Staphylococcus aureus* [19], *Streptococcus mutans* [19], *Enterococcus faecalis* [19], *Escherichia coli* [19] bacteria, and *Candida albicans* [19] fungus has been demonstrated. The different methodologies and aims of the studies made it impossible to compare them. The antibacterial effect of ZnO was also proven in a study by Hammad et al. [23], which used NiTi arches in orthodontics coated with ZnO nanoparticles. Such surface modification has been proven to exert antibacterial activity against strains of *Staphylococcus aureus*, *Streptococcus pyogens*, and *Escherichia coli*.

Noorollahian et al. [17] did not evaluate whether the ZnO-containing coating itself exhibits antibacterial properties. However, the combination of this method with the use of the antibiotic doxycycline has been investigated. This combination proved effective in preventing the growth of *Porphyromonas gingivalis* for up to 30 days. Another study in which an antibiotic was used to enhance antibacterial properties is the study conducted by Anggani [16], rated High RoB. This study proved the greatest antibacterial potential against *Porphyromonas gingivalis* for the group using azithromycin alone, while the other methods (chitosan, chitosan with azithromycin) also showed antibacterial properties, but to a lesser extent.

In a study by Bahrami [21], ZnONPs-coated mini-screws impregnated with polymicrobial biofilm irrigated with PBS were studied, as well as the effect of their subsequent exposure to LED irradiation, ultrasound waves, and a combination of the latter two. Antibacterial activity against *Porphyromonas gingivalis*, *Prevotella intermedia*, and *Aggregatibacter actinomycetemcomitans* was obtained in each of the tested groups. The best result was obtained in the group where both methods were used, and also surprisingly in the group in which uncoated microimplants were immersed in 0.2% CHX solution for 5 min. This study points to the possibilities offered by ZnO coating, as well as exposure methods that improve antibacterial properties, such as LED irradiation and ultrasound waves.

The use of chlorhexidine and its antibacterial properties seems to be an interesting method. Often, gels or rinses containing this antiseptic are recommended as post-operative recommendations for patients in Poland. In a study by Chin et al. [24], the authors found that chlorhexidine gluconate and sodium fluoride exhibited significant antibacterial and plaque-forming activity on the surface of the microimplants, demonstrating their important role in oral hygiene in orthodontic patients.

Another substance that was often used in research was chitosan. Chitosan is considered to be a non-toxic, biocompatible, and biodegradable compound [25,26], and due to its versatile effects, including antibacterial, antifungal, analgesic, and anticancer, it is finding more and more applications in the cosmetic industry, medicine, or biomedical applications [27], such as wound healing, controlled drug delivery, and tissue regeneration [28]. It was used in the study by Alhazmi [18] (Moderate RoB), Anggani [16], and Sreenivasagan [14] (both High RoB), so the results obtained should be approached with caution. In the previously mentioned study by Anggani [16], an antibacterial effect was obtained with chitosan alone, chitosan with azithromycin, and azithromycin alone. In a study by Sreenivasagan [14], chitosan-based silver-impregnated nanoparticles were used, which also showed antibacterial properties but poor antifungal values. In a study by Alhazmi [18], the best antibacterial effect was obtained when chitosan was used in combination with hydroxyapatite. In these studies, efficacy against *Streptococcus mutans* [14,18], *Streptococcus sanguis* [18], *Streptococcus salivarius* [18], *Enterococcus faecalis* [18], *Porphyromonas gingivalis* [16], *Staphylococcus aureus* [14], *Lactobacillus* [14], and *Candida albicans* [14] was demonstrated. A study that also tested the antibacterial properties of chitosan was conducted by Ly et al. [29]. This paper was not qualified for review because the authors used titanium alloy discs for the study. However, the authors were able to prove that reduced biofilm formation of *Streptococcus mutans* and *Streptococcus sobrinus* was achieved with samples containing chitosan. The above studies show that chitosan is a promising option for modifying the surface of microimplants to impart antimicrobial properties, particularly when used in combination with another substance with similar effects.

In the study conducted by Alhazmi [18], hydroxyapatite was used. This substance was also used in the study [19], rated Low RoB. Both studies showed efficacy against *Streptococcus mutans* and *Enterococcus faecalis* bacteria. Both studies testify to the potential for HA to be used for antibacterial properties, particularly when used in combination with another substance. Similar results were obtained by Abdulkareem [30], who studied the antibacterial properties of three types of coatings (nano-ZnO; nZnO and nHA; and nHA) with which he coated titanium plates. All coatings have shown antibacterial activity. A study by Leelanarathiwat [31] tested the combination of antimicrobial activity for hydroxyapatite–tryptophan complex with Gray Titania coating and photocatalysis on titanium alloy substrate. The study was conducted on *Porphyromonas gingivalis*, *Tannerella forsythia*, and *Aggregatibacter actinomycetemcomitans*. The results showed that the photoactivated hydroxyapatite–tryptophan complex and Gray Titania as a photocatalytic coating have antibacterial effects; however, the coating itself did not show antibacterial effects against bacteria involved in the formation of peri-implantitis. The above studies indicate the possible use of HA, especially in combination with another method, to increase the antibacterial properties of the coating.

In the study conducted by Abo-Elmahasen [19], silver was used in addition to HA, yielding antibacterial properties. Many other studies confirm this result for various bacterial species, *Streptococcus mutans* and *Porphyromonas gingivalis* [32], or those less commonly present in the oral cavity—such as *Staphylococcus aureus* [33,34] or *Escherichia coli* [34]. The effect of this element on antibacterial properties was also studied by Venugopal [13] and Subramanian [15]. Venugopal et al. [13] evaluated a Moderate RoB, proving an antibacterial effect occurring only with AgNP-coated biopolymer. In contrast, no such properties were found for regular Ag-NPs. This study shows that it is not only the substance used that matters but also the form in which it is used. Both studies demonstrated the effectiveness of silver-containing coatings on *Streptococcus mutans* bacteria.

In the work of Subramanian [15], rated High RoB, it was proven that a coating containing AgNPs as well as SeNPs showed antibacterial properties, greater in the former case. In both cases, a biopolymer was used.

Studies show that SeNPs not only have antibacterial effects against *Escherichia coli* and *Staphylococcus aureus* [35] but also against *Porphyromonas gingivalis*, a bacterium commonly associated with peri-implantitis [36]. The antimicrobial effect of SeNPs was also described, in which SeNPs were shown to inhibit macrophage proliferation, thereby reducing the inflammatory response around implants [35].

Another way to impart antimicrobial properties to microimplants is to treat the microimplant surface with PEG polymerization plasma, as demonstrated in the study conducted by Rodriguez-Fernandez [20], rated as a high-quality study. All tested PEG samples showed decreased bacterial adhesion, either for *Streptococcus sanguinis* or *Lactobacillus salivarius*. This result confirms a study by Buxadera-Palomero et al. [37], who also proved reduced adhesion of *Streptococcus sanguinis* and *Lactobacillus salivarius* to PEG-like coating on the titanium surface. In addition, it has been shown that despite reducing bacterial adhesion to the surface, the coating does not affect fibroblast and osteoblast adhesion [37].

### Limitations

All the studies analyzed in the review are in vitro studies. To date, there is no sufficiently large number of pertinent studies conducted on humans. Another problem is the diversity in testing methodology and the inability to compare all the methods described, which makes it difficult to assess the antibacterial properties of the coating.

## 5. Conclusions

- All of the microimplant surface modifications analyzed showed antibacterial properties. The presented methods, such as the use of ZnO, antibiotics, chlorhexidine, silver compounds, selenium, hydroxyapatite, and PEG polymerization plasma, are an intriguing possibility for improving the properties of orthodontic microimplants, and thus reducing the risk of complications in the form of local inflammation. However, because of the still small number of studies on the subject and different methodologies, more studies are needed to assess the effectiveness of the given methods.
- In vitro studies are required to enable the implementation of new technology in the orthodontic treatment of patients.

## Figures and Tables

**Table 1:** Summary of findings, including type of coating, bacteria used, and results of the study.

	Author, Year of Publication	Type of Coatings Tested	Bacteria Used in the Study	Results and Conclusions of the Study
1.	Venugopal 2017 [13]	Ti-AgNP (regular silver nanoparticles)	*Streptococcus mutans*	Ti-AgNP—not effective.
			*Streptococcus sanguinis*	
		Ti-BP-AgNP (biopolymer AgNP)	*Aggregatibacter actinomycetemcomitans*	Ti-BP-AgNP—excellent antibacterial properties.
2.	Sreenivasagan 2020 [14]	Chitosan-based silver-impregnated nanoparticles	*Streptococcus mutans*	Strong antibacterial activity was observed against *Staphylococcus aureus*, *Lactobacillus*, antibacterial activity was observed against Streptococcus mutans, but the zone of inhibition was less than that observed in the other bacteria. The synthesized nanoparticles did not have a very strong antifungal activity.
			*Staphylococcus aureus*	
			*Lactobacillus*	
			*Candida albicans*	
3.	Subramanian 2021 [15]	Ti-BP-AgNP (biopolymer with silver nanoparticles)	*Streptococcus mutans*	Ti-BP-AgNP and Ti-BP-SeNP showed strong antibacterial activity against *Lactobacillus* and *Staphylococcus aureus.* Antibacterial activity against *Streptococcus mutans* was slightly less than observed in other bacteria. Ti-BP-SeNP-coated mini-implants show less antibacterial activity, but the difference is marginal when compared to AgNP.
			*Staphylococcus aureus*	
		Ti-BP-SeNP (biopolymer with selenium nanoparticles)	*Lactobacillus*	
4.	Anggani 2021 [16]	Chitosan	*Porphyromonas gingivalis*	Orthodontic mini-implants coated with chitosan, chitosan with azithromycin, or azithromycin only effectively suppressed *Porphyromonas gingivalis* biofilm formation.
		Chitosan+ azithromycin		
		Azitrthomycin		
5.	Noorollahian 2022 [17]	Nanotubes	*Porphyromonas gingivalis*	Nanotubes—not effective alone.
		ZnO (zinc oxide) doped into nanotubes + doxycycline		Nanotubes containing doped ZnO and doxycycline—capable of preventing bacterial growth around mini-implant surfaces.
6.	Alhazmi 2022 [18]	Hydroxyapatite Chitosan	*Streptococcus sanguis*	Both hydroxyapatite and chitosan nanoparticles have shown antibacterial properties.
			*Streptococcus mutans*	
			*Streptococcus salivarius*	Hydroxyapatite showed enhanced antibacterial activity and more obvious damage in the bacterial cell membrane than that of synthesized chitosan nanoparticles.
			*Enterococcus faecalis*	
7.	Abo-Elmahasen 2022 [19]	Ag/HA NPs (silver/hydroxyapatite nanoparticles)	*Enterobacter aeruginosa*	Orthodontic mini-screws (OMSs) coated with ZnO or Ag/HA NPs demonstrated clear antimicrobial activity against all the investigated microorganisms.
			*Staphylococcus aureus*	
			*Streptococcus mutans*	
		ZnO NPs (zinc oxide nanoparticles)	*Enterococcus faecalis*	ZnO NP-coated OMS had the highest antimicrobial activity than Ag/HA-coated OMS.
			*Escherichia coli*	
			*Candida albicans*	
8.	Rodriguez-Fernandez 2022 [20]	PEG (polyethylene glycol) polymerization plasma	*Spectrococcus sanguinis*	All tested PEG samples showed decreased bacterial adhesion.
			*Lactobacillus salivarius*	
9.	Bahrami 2023 [21]	0.2% CHX ZnONPs-coated mini-screws aPDT ^1^ aSDT ^2^ aPSDT ^3^	*Porphyromonas gingivalis*	A significant reduction in log_10_ CFU/mL of periopathogens was observed in groups treated with aPDT, aSDT, aPSDT, and 0.2% CHX up to 6.81, 6.63, 5.02, and 4.83 log, respectively, when compared with control groups. The current results suggest that ZnONPs-mediated aPSDT could have the greatest antimicrobial efficacy in reducing the periopathogenic biofilms around the mini-screw surface.
			*Prevotella intermedia*	
			*Aggregatibacter actinomycetemcomitans*	
10.	Othman 2024 [22]	TiO_2_ NPs (titanium dioxide nanoparticles) TiO_2_ +ZnO NP (titanium dioxide with zinc oxide)	*Staphylococcus aureus*	The antibacterial activity of micro-implants coated with TiO_2_ or TiO_2_ZnO NPs showed greater antibacterial activity in comparison with the control.
			*Streptococcus mutans*	
			*Porphyromonas gingivalis*	

^1^ ZnONPs-coated mini-screws impregnated with polymicrobial biofilm were exposed to the LED irradiation for one min; ^2^ ZnONPs-coated mini-screws impregnated with polymicrobial biofilm were exposed to the ultrasound waves irradiation for one min; ^3^ ZnONPs-coated mini-screws impregnated with polymicrobial biofilm were exposed to the LED irradiation for one min and immediately ultrasound waves irradiation for one min.

**Table 2:** Risk of Bias assessment.

		Venugopal 2017 [13]	Sreenivasagan 2020 [14]	Subramanian 2021 [15]	Anggani 2021 [16]	Noorollahian 2022 [17]	Alhazmi 2022 [18]	Abo-Elmahasen 2022 [19]	Rodriguez-Fernandez 2022 [20]	Bahrami 2023 [21]	Othman 2024 [22]
**Introduction**	Has the aim of the study been defined?	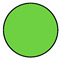	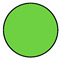	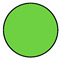	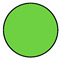	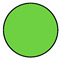	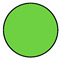	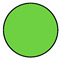	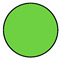	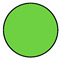	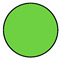
**Methods**	Is it described where the mini-implants for the study were obtained from?	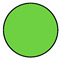	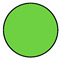	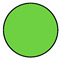	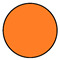	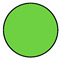	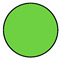	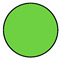	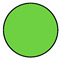	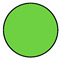	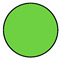
	Are the properties of the microimplants described, in particular the material from which they are made?	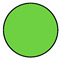	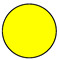 ^1^	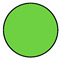	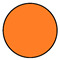	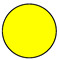 ^2^	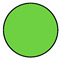	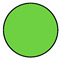	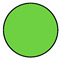	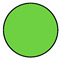	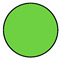
	Were the mini-implants used in the study cleaned before coating/sterile?	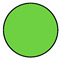	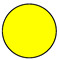	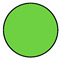	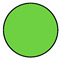	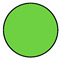	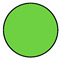	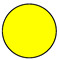	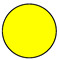	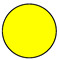	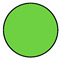
	Was the manner in which the study coatings were created described in detail?	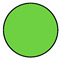	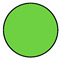	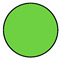	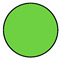	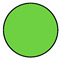	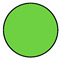	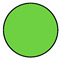	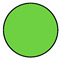	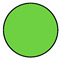	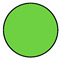
	Was the composition of the coatings described?	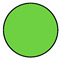	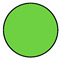	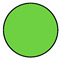	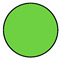	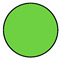	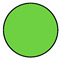	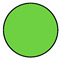	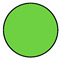	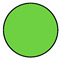	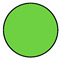
	Was the accuracy of the surface coating of the mini-implants examined?	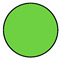	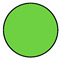	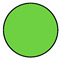	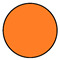	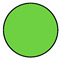	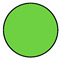	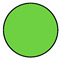	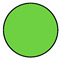	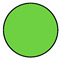	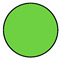
	Were the conditions under which the number of bacteria was studied described?	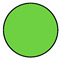	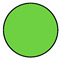	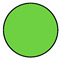	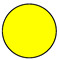	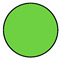	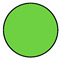	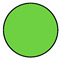	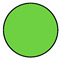	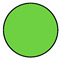	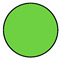
	Was it verified that the coating was not cytotoxic?	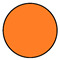	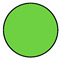	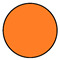	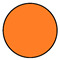	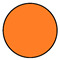	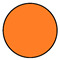	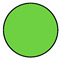	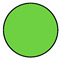	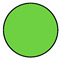	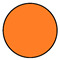
	Was the study conducted on human cells?	**-**	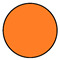	**-**	**-**	**-**	**-**	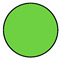	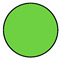	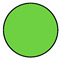	**-**
**Results**	Was the study repeated to check the results more than once? Or was more than 1 sample tested?	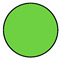	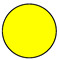	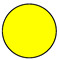	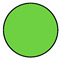	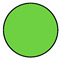	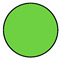	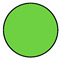	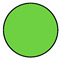	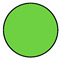	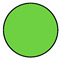
	Was a statistical analysis of the results performed?	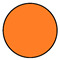	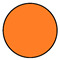	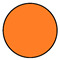	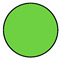	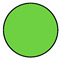	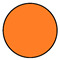	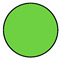	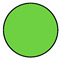	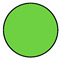	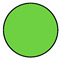
	No external funding?	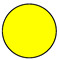	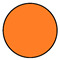	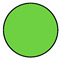	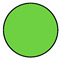	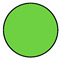	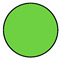	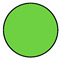	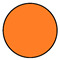	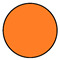	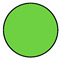
	Were the results presented clearly and transparently?	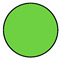	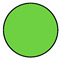	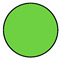	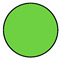	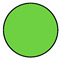	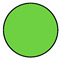	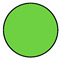	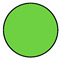	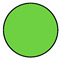	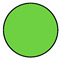

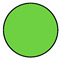
 Yes/Low risk; 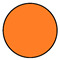 No/High Risk of Bias; 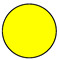 Not known/not mentioned. ^1^ The authors only wrote that the mini-implants were made of titanium—too little information about the composition. ^2^ The authors described the size and the company from which the microimplants were purchased, but there is no information on the composition of the alloy from which they were made.

**Table 3:** Summary of Risk of Bias assessment.

Risk of Bias	Studies	Main Factors Increasing the Quality of the Study	Main Factors Decreasing the Quality of the Study
Low	Abo-Elmahsen [19]	These authors performed a full analysis of the cytotoxicity of the tested coatings, and what is more, they did it with human-derived cells. These papers stand out due to the evaluation of cytotoxicity on human cells, as well as the statistical analysis of the results obtained.	Lack of information regarding the cleaning of microimplants before the surface modification.
	Rodriguez-Fernandez [20]		Funding. Lack of information regarding the cleaning of microimplants before the surface modification.
	Bahrami [21]		Funding. Lack of information regarding the cleaning of microimplants before the surface modification.
Moderate	Alhazmi [18]	Cleaning of microimplants before the surface modification.	Lack of statistical analysis of the results obtained. Lack of cytotoxicity verification.
	Othman [22]	Cleaning of microimplants before the surface modification Statistical analysis of the results obtained.	The only factor that influenced the assessment of this paper was the lack of cytotoxicity verification in their study. The authors of this systematic review concluded that since this factor significantly affects the level of inflammation, the lack of cytotoxicity studies forces a reduction in the quality of the study.
	Noorollahian [17]	The authors showed the greatest care of all in cleaning the surface of the microimplants, as they did so both before and after modifying the surface.	Lack of an accurate description of the microimplants used for the study, particularly the material from which they were made.
	Venugopal [13]	Cleaning of microimplants before the surface modification.	Lack of statistical analysis of the results obtained. Lack of cytotoxicity verification.
High	Anggani [16]	Statistical analysis of the results obtained.	Lack of description regarding where the microimplants used in the study came from, as well as the lack of information regarding the material. This is the only study that did not evaluate the accuracy of the surface modification method used.
	Subramanian [15]	Description of material the microimplants were made of.	Lack of statistical analysis of the results obtained. Lack accurate information about the size of the study groups, whether more than one sample was used in the study, or whether the study was repeated to verify the results obtained.
	Sreenivasagan [14]	Testing cytotoxicity using shrimp culture.	Lack of statistical analysis of the results obtained. Lack accurate information about the size of the study groups, whether more than one sample was used in the study, or whether the study was repeated to verify the results obtained. No description where the microimplants used in the study came from or exactly what alloy they were made of.

## Data Availability

No new data were created or analyzed in this study. Data sharing is not applicable to this article.

## Supplementary Materials

The following supporting information can be downloaded at: https://www.mdpi.com/article/10.3390/ma18153575/s1, Supporting File: PRISMA Checklist.
